# Supplementary material for: Hybrid Resistance and Virulence Plasmids in “High-Risk” Clones of Klebsiella pneumoniae, Including Those Carrying blaNDM-5
Source: Microorganisms. 2019 Sep 6;7(9):326. doi: 10.3390/microorganisms7090326 (PMC6780558; doi:10.3390/microorganisms7090326)
Supplement: Supplementary file 1 [file microorganisms-07-00326-s001.pdf]

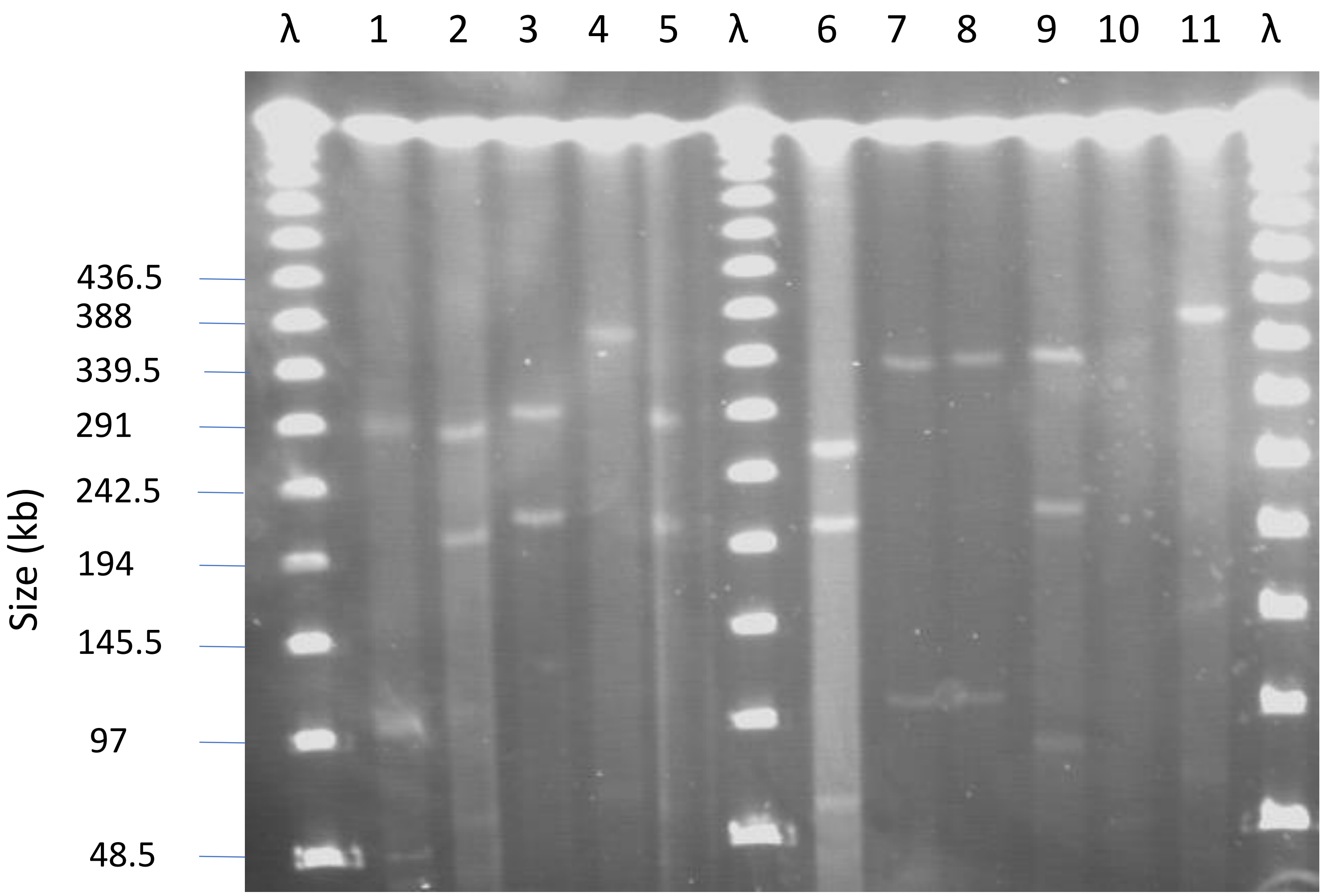

Figure S1. Examples of S1-PFGE results. Plasmids were sized against CHEF λ ladder. Isolates were: lane 1, Kpv_ST383_L2; lane 2, Kpv_ST101_OXA-48; lane 3, Kpv_ST48_NDM; lane 4, Kpv_ST383_NDM_OXA-48; lane 5, Kpv_ST101_SE2_2; lane 6, Kpv_ST101_L5; lane 7, Kpv_ST147B_SE1_1; lane 8, Kpv_ST147_L3; lane 9, Kpv_ST15_NW1; lane 10, KpvST147L_NDM; lane 11, Kpv_ST383_S1.

Table S1. Results of MIC determination (in mg/L) for 22 antibiotics/antibiotic combinations on seven isolates carrying hybrid virulence and resistance plasmids. Results were interpreted using EUCAST breakpoints (version 9.0) (http://www.eucast.org/fileadmin/src/media/PDFs/EUCAST_files/Breakpoint_tables/v_9.0_Breakpoint_Tables.pdf). Resistance is indicated in red, Susceptible, increased exposure in amber and Susceptible in green.

Antibiotic	KpvST101_OXA-48	Kpv_ST101_SE2_2	Kpv_ST383_L2	Kpv_ST383_S1	Kpv_ST147_SE1_2	KpvST147L_NDM	Kpv_ST15_NDM
Amikacin	>64	>64	>64	>64	>64	>64	>64
Gentamycin	>32	>32	>32	>32	>32	>32	>32
Tobramycin	>32	>32	>32	>32	>32	>32	>32
Ampicillin	>64	>64	>64	>64	>64	>64	>64
Aztreonam	>64	64	>64	>64	>64	>64	64
Cefepime	32	32	>64	>64	64	64	>64
Cefotaxime	256	256	>256	>256	>256	256	>256
Cefoxitin	64	64	>64	>64	>64	>64	>64
Ceftazidime	32	128	>256	>256	>256	>256	>256
Ertapenem	>16	>16	>16	>16	>16	>16	>16
Imipenem	2	2	64	64	32	16	32
Meropenem	8	16	>32	>32	16	32	>32
Piperacillin/ tazobactam	>64	>64	>64	>64	>64	>64	>64
Temocillin	>128	>128	>128	>128	128	128	128
Cefotaxime/ cloxacillin	256	128	>256	>256	>256	256	>256
Ceftolozane/ tazobactam	>16	>16	>16	>16	>16	>16	>16
Ceftazidime/ avibactam	0.125	0.125	>32	>32	>32	>32	>32
Colistin	≤0.5	≤0.5	32	1	≤0.5	1	≤0.5
Ciprofloxacin	>8	>8	>8	>8	>8	>8	>8
Minocycline	4	4	32	not tested	8	not tested	32
Tigecycline	1	1	0.5	4	1	2	4
Fosfomycin	not tested	not tested	32	16	not tested	not tested	16
